# Magnonic Metamaterials for Spin-Wave Control with Inhomogeneous Dzyaloshinskii–Moriya Interactions

**DOI:** 10.3390/nano12071159

**Published:** 2022-03-31

**Authors:** Fengjun Zhuo, Hang Li, Zhenxiang Cheng, Aurélien Manchon

**Affiliations:** 1 School of Physics and Electronics, Henan University, Kaifeng 475004, China; fengjun.zhuo@kaust.edu.sa; 2 Physical Science and Engineering Division (PSE), King Abdullah University of Science and Technology (KAUST), Thuwal 23955-6900, Saudi Arabia; 3 Institute for Superconducting and Electronic Materials, Australian Institute of Innovative Materials, Innovation Campus, University of Wollongong, Squires Way, North Wollongong, NSW 2500, Australia; 4 Aix Marseille University, CNRS, CINAM, 13288 Marseille, France

**Keywords:** spin waves, Dzyaloshinskii–Moriya interaction, ferromagnetism, spintronics

## Abstract

A magnonic metamaterial in the presence of spatially modulated Dzyaloshinskii–Moriya interaction is theoretically proposed and demonstrated by micromagnetic simulations. By analogy to the fields of photonics, we first establish magnonic Snell’s law for spin waves passing through an interface between two media with different dispersion relations due to different Dzyaloshinskii–Moriya interactions. Based on magnonic Snell’s law, we find that spin waves can experience total internal reflection. The critical angle of total internal reflection is strongly dependent on the sign and strength of Dzyaloshinskii–Moriya interaction. Furthermore, spin-wave beam fiber and spin-wave lens are designed by utilizing the artificial magnonic metamaterials with inhomogeneous Dzyaloshinskii–Moriya interactions. Our findings open up a rich field of spin waves manipulation for prospective applications in magnonics.

## 1. Introduction

Magnonics (or magnon spintronics) is an emerging field concentrating on the generation, detection and manipulation of magnons, the quanta of spin-wave, in ferromagnetic or antiferromagnetic metals and insulators [1,2,3,4,5,6,7,8,9]. As spin waves in magnetic insulators exhibit both low energy dissipation and long coherence length, these constitute a competitive alternative to electronic devices and are deemed to be a promising candidate as a high-quality information carrier [10,11,12,13]. Over the past decades, many properties of spin waves have been demonstrated experimentally, in analogy with electromagnetic waves: excitation and propagation [14,15,16,17,18], reflection and refraction [19,20,21,22], interference and diffraction [23,24,25] and tunneling and the Doppler effect [26,27,28].

Thus, far, based on recent progress in the fabrication of magnetic nanostructures, various device concepts have been proposed, such as spin-wave logic gates and circuits [10,29,30], waveguides [31,32], multiplexors [33], splitter [34] and diodes [35]. The implementations of those devices is usually achieved by the application of external local magnetic fields [26], spin current [28,36] and magnetic textures (for example, the chiral domain wall) [29,31,37] to control the dispersion relation of spin waves, thereby, steering the spin-wave propagation properties. Despite the soundness of the concepts, however, there are some inherent drawbacks and obstacles to applications. First, generating a local high-frequency magnetic field on micro-sized devices complicates the structure design, and the local field is often spatially inhomogeneous, which can inhibit the benefits of the device [38]. In addition, unstable magnetic textures under external excitation and at room temperature may give rise to poor reliability and high bit-error rates. Therefore, it is desirable to find a new method to manipulate the propagation of spin waves.

Recent discoveries in graded-index magnonics and magnonic metamaterials provide a new way to manipulate spin-wave propagation [39,40], which is inspired by the fields of graded-index photonics (or photonic metamaterials) [41,42,43]. The core idea of graded-index magnonics is to manipulate spin-wave propagation by designing a spatially varied magnonic refractive index. In magnetic thin films with in-plane magnetization, the spin-wave dispersion relation described by the Landau–Lifshitz–Gilbert (LLG) equation exhibits a much more complex structure compared to the isotropic dispersion relation of light. This offers extremely rich opportunities to modulate the magnonic refractive index.

Up to now, it has been shown that the graded magnonic refractive index can be created by modification of the material properties, such as non-uniform saturation magnetization or exchange constant [44,45,46,47], the magnetic anisotropy [19,20] or the internal magnetic field [37,48]. This index can be also achieved by utilizing a non-uniform external magnetic field [39,49,50,51], electric field (voltage) [52,53] or temperature [54,55]. Therefore, graded-index magnonics are expected to overcome the current limitation of magnonics and pave feasible routes for the implementation of spin-wave devices.

In this paper, we theoretically propose a magnonic metamaterial, in which we modulate the refractive index of spin waves with the inhomogeneous Dzyaloshinskii–Moriya interaction (DMI) to avoid a barely controllable local magnetic field and unstable magnetic textures. The DMI is an antisymmetric exchange interaction arising from the lack of structural inversion symmetry in magnetic films [56,57]. It has been found both for bulk materials [58,59,60] and magnetic interfaces [61].

Here, we focus on a spatial inhomogeneous interfacial DMI present in ferromagnet/heavy metal (FM/HM) bilayers realized by tuning the thickness of ferromagnetic layer or HM layer [62,63,64,65], the degree of hybridization between 3d-5d states [66] or utilizing a local gating [67]. We begin our work by rapidly deriving the spin-wave dispersion relation with spatially modulated DMI. Then, we further study spin-wave refraction and reflection at the interface between two magnetic media with different DMI and build a generalized Snell’s law of spin waves, similar to Snell’s law in optics.

According to the magnonic Snell’s law, spin-wave can also experience total internal reflection (TIR) at the DMI step interface when their incident angle is larger than a critical value (i.e., the critical angle). Moreover, magnonic Snell’s law and TIR are observed and confirmed by micromagnetic simulations. Utilizing the artificial magnonic metamaterials based on spatially modulated DMI, a spin-wave fiber owing to TIR (which can transmit spin waves over a long distance) and a spin-wave lens holding tremendous possibility to build spin-wave circuits are proposed as proofs of concept.

The paper is organized as follows. In Section 2, we introduce our theoretical model and method. Detailed results of micromagnetic simulations are presented in Section 3. Then, we discuss the realization of spin-wave fibers and lenses in Section 4. Finally, we end the paper with a summary in Section 5.

## 2. Analytical Model

### 2.1. Magnonic Snell’s Law

**Figure 1:**
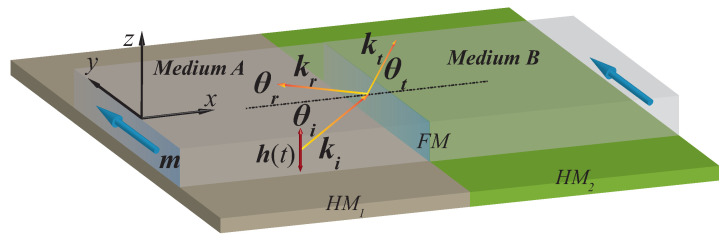
Schematic illustration of spin-wave transmission and reflection at an interface between media A and B with different interfacial DMI in a thin YIG film. The interfacial DMI step here is realized by utilizing two different HM layers (HM${}_{1}$ and HM${}_{2}$) below the YIG film. The blue arrows along the $\hat{y}$ direction denote the magnetization m. $k_{i}$, $k_{t}$ and $k_{r}$ are the wave vectors of the incident, refracted and reflected spin-wave shown as the yellow and red arrows, respectively. $\theta_{i,t,r}$ denote their angles with respect to the interface normal. The red double-headed arrow shows the Gaussian distribution AC Magnetic field h(t) exciting the spin-wave.

We consider a thin magnetic film in the x−y plane with the thickness much smaller than lateral dimensions of the film ($L_{z}\ll L_{x},L_{y}$), whose initial magnetization is homogeneous along the $\hat{y}$ direction. The magnetization dynamics are governed by the LLG Equation [68],

(1) $$\frac{\partial m}{\partial t}=-\frac{\gamma }{M_{s}}m\times H_{eff}+\alpha m\times \frac{\partial m}{\partial t},$$

where m is the unit direction of local magnetization $M=M_{s}m$ with a saturation magnetization $M_{s}$. α is the phenomenological Gilbert damping constant, and γ is the gyromagnetic ratio. Here, $H_{eff}=A^{*}\nabla^{2}m-D^{*}\left(x\right)\left(\hat{z}\times \nabla \right)\times m-K^{*}m_{y}\hat{y}$ is the effective field [69], and $A^{*}=2A/\mu_{0}M_{s}$, $D^{*}\left(x\right)=2D\left(x\right)/\mu_{0}M_{s}$, $K^{*}=2K/\mu_{0}M_{s}$. *A* is the symmetric exchange constant, D(x) is the interfacial antisymmetric DMI constant spatially inhomogeneous along the *x* direction, *K* is the in-plane anisotropy and $\mu_{0}$ is the permeability of vacuum. Under the perturbative approximation, the small-amplitude spin waves propagating in the x−y plane take the following form [70]:

(2) $$m=\hat{y}+\delta m\exp\left[i\left(\hat{k}\cdot \hat{r}-wt\right)\right],$$

where $\delta m=(\delta m_{x},0,\delta m_{z})$ is the spin-wave contribution to magnetization (|δm|≪1). $\delta k=(k_{x},k_{y},0)$ is the spin-wave wavevector. Considering the system shown in Figure 1, we use a DMI step (i.e., $D=D_{1}$ in medium A and $D=D_{2}$ in medium B) to induce a difference in spin-wave dispersion relations between two magnetic domains. Inserting Equation (2) into Equation (1) and neglecting higher order terms, we obtain the spin-wave dispersion relation in each region [71,72],

(3) $$\omega \left(k_{n}\right)=\gamma \mu_{0}\left(K^{*}+A^{*}k_{n}^{2}-D_{n}^{*}k_{n,y}\right),$$

with $D_{n}^{*}=2D_{n}/\mu_{0}M_{s}$ and $k_{n}=\sqrt{k_{n,x}^{2}+k_{n,y}^{2}}$. The spin-wave group velocity is $v_{g,n}=\partial \omega /\partial k_{n}=2A^{*}k_{g,n}$, where $k_{g,n}=k_{n}-\delta_{n}\hat{y}$ and $\delta_{n}=D_{n}^{*}/2A^{*}$. To simplify the model, we assume that the group velocity is parallel to the phase velocity at each point of the dispersion relation—that is to say, the dispersion relation is isotropic.

**Figure 2:**
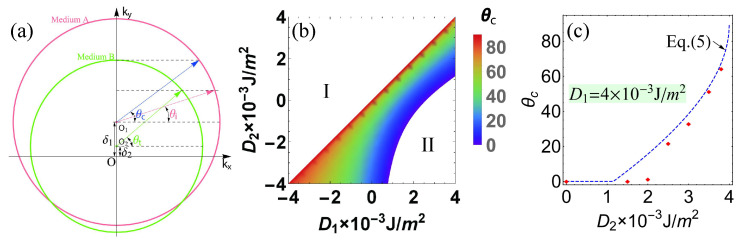
(**a**) Schematic illustrations of reflection and refraction of spin-wave at an interface between two different media in wave vector ($k_{x}-k_{y}$) space. The pink and green circles indicate the individual frequency contours of the allowed modes in the same-color-coded media A and B, respectively. The color-coded arrows denote the spin-wave vectors k propagating in each medium, as indicated by the incident (pink) and refracted (green) rays. The blue arrow denotes the critical angle. (**b**) Phase diagrams of critical angle $\theta_{C}$ in the $D_{1}-D_{2}$ plane. No TIR exists in the white regions. (**c**) Critical angle $\theta_{c}$ as a function of DMI constants $D_{2}$ with a fixed DMI constant $D_{1}=4\times 10^{-3}$ J/m${}^{2}$. The symbols (red squares) are simulation data, and the solid curve represents the analytical results of Equation (5).

Equation (3) represents an isofrequency circle with radius $k_{g,n}$ in momentum space, whose center deviates from the origin by $\delta_{n}$ in $-\hat{y}$ direction as illustrated in Figure 2a. Nevertheless, in the magnetic films with in-plane magnetization, the spin-wave dispersion relation is anisotropic at low frequencies, where dipolar contribution dominates. When increasing frequency, the isofrequency contours smoothly transform through elliptical to almost circular. Consequently, the dispersion relation is isotropic as determined by the exchange interactions at high frequencies. In the following simulations, we use quite high frequency spin waves (100 GHz), and thus the spin-wave dynamic is determined by the exchange interactions.

Based on translation symmetry considerations, the refraction angle obeys the generalized Snell’s law, which guarantees continuity of the tangential components of the k vector across the DMI step interface along the $\hat{y}$ axis, such that $k_{i,y}=k_{t,y}$ [19,20,37]. Consequently, the generalized magnonic Snell’s law based on modifying the dispersion relation with inhomogeneous DMI can be rewritten in the following form:

(4) $$k_{g,i}\sin\theta_{i}+\delta_{i}=k_{g,t}\sin\theta_{t}+\delta_{t},$$

where $k_{g,n}=\sqrt{\left(\omega /\gamma \mu_{0}-K^{*}\right)/A^{*}+\delta_{n}^{2}}$ is the value of $k_{g,n}$. Here, the generalized Snell’s law shown in Equation (4) is derived for an interface between two spin-wave media with different material parameters (interfacial DMI), which can be viewed as graded-index magnonic metamaterials. However, this is different from Snell’s laws based on the interface inside magnetic textures, such as chiral domain walls (the interface formed by two opposite magnetic domains) [37].

### 2.2. Total Internal Reflection

Analogously to the case of electromagnetic waves in photonics or acoustic waves in phononics, spin waves are also expected to be completely reflected by the interface when a spin-wave travels from a denser medium with a higher refractive index to a thinner medium with a lower refractive index known as TIR. TIR occurs when the incident angle $\theta_{i}\geq \theta_{c}$, where $\theta_{c}$ is often called the critical angle. When $\theta_{i}=\theta_{c}$, the refracted spin-wave travels along the interface between the two media or the angle of refraction $\theta_{t}$ is π/2. According to the magnonic Snell’s law in Equation (4), the critical angle can be expressed as

(5) $$\theta_{c}=\arcsin\left(\frac{k_{g,t}-\delta }{k_{g,i}}\right),$$

where $\delta =\delta_{i}-\delta_{t}$. Specifically, Equation (5) shows that $\theta_{c}$ equals π/2 when the DMI is homogenous ($D_{1}=D_{2}$), i.e., all incident spin waves are fully transmitted and no reflection occurs. Furthermore, when δ (the difference between DMI in two regions) is chosen to be large enough, a gap falls in between the two isofrequency circles and TIR occurs at all incident angles (i.e., $\theta_{c}=0$). Equations (4) and (5) are the main analytical results in our paper.

## 3. Micromagnetic Simulations

To test the validity of these analytical findings in realistic situations, micromagnetic simulations have been proven to be an efficient tool for the investigation of spin-wave dynamics in various magnetic textures and geometries. The simulations here are performed in the GPU-accelerated micromagnetic simulations program MuMax3 [73], which solves the time-dependent LLG Equation (1) based on the finite difference method. In our simulations, we used typical magnetic parameters for YIG at zero temperature [74]: $M_{s}=0.194\times 10^{5}$ A/m, A=3.8 pJ/m and $K=10^{4}$ J/m${}^{3}$.

All simulations presented here were performed for a thin film of size $L_{x}\times L_{y}\times L_{z}$, which discretized with cuboid meshes of dimensions $l_{x}\times l_{y}\times l_{z}$. The lateral dimensions of unit mesh ($l_{x}$ and $l_{y}$) and the thickness of the film $L_{z}$ are all smaller than the exchange length of YIG [3]. The simulations were implemented with the mesh size 2×2×2 nm${}^{3}$. The simulations were split into two stages: the static and dynamic stage. In the first stage, the static stage, the magnetic configuration is stabilized by minimization of the total energy starting from the random magnetic configuration with a high value of damping (α=0.5).

In the dynamic stage of the simulations, the equilibrium magnetic configuration was used to excite a spin-wave beam that propagates through the film with a small damping parameter (α=0.0005) to ensure long-distance propagation. During this step, a Gaussian type spin-wave beam was continuously generated by a harmonic dynamic external magnetic field following a Gaussian distribution function in a small rectangular region (red double-headed arrow shown in Figure 1). The detailed description of the Gaussian spin-wave beam generation procedure can be found in Refs. [75,76,77].

The Gaussian spin-wave beam is clearly visible and does not change with time after continuously exciting a sufficiently long time, which corresponds to a steady spin-wave propagation. Moreover, to avoid spin-wave reflection at the boundaries of the film, absorbing boundary conditions are applied on all boundaries by assigning a large damping constant (α=1) near the edges.

In order to verify the magnonic Snell’s law in Equation (4) for the spin-wave propagation through a DMI step interface, we focus on a 4 μm × 4 μm × 2 nm nanowire. The spin-wave beams presented here are all exchange-dominated spin waves with 200 nm beam width and 30 nm wavelength generated by an external AC magnetic field with frequency f=100 GHz. The phase diagram of the critical angle $\theta_{c}$ in the $D_{1}-D_{2}$ plane is shown in Figure 2b. As $D_{1}<D_{2}$ in the white region I, spin waves transmit from a thinner medium with a lower refractive index to a denser medium with a higher refractive index, and thus no TIR happens.

A gap falls in between the two isofrequency circles—in other words, TIR occurs in all incident angles when δ is chosen to be large enough as shown in the white region II. Figure 2c shows the critical angle as a function of the DMI constant $D_{2}$ in medium B, where the DMI constant of medium A is fixed at $D_{1}=4\times 10^{-3}$ J/m${}^{2}$. All incident angle spin waves are totally reflected at a small $D_{2}$ corresponding to Region II in Figure 2b. After that, the critical angle increases monotonically with $D_{2}$ and shows a good agreement with the analytical results.

**Figure 3:**
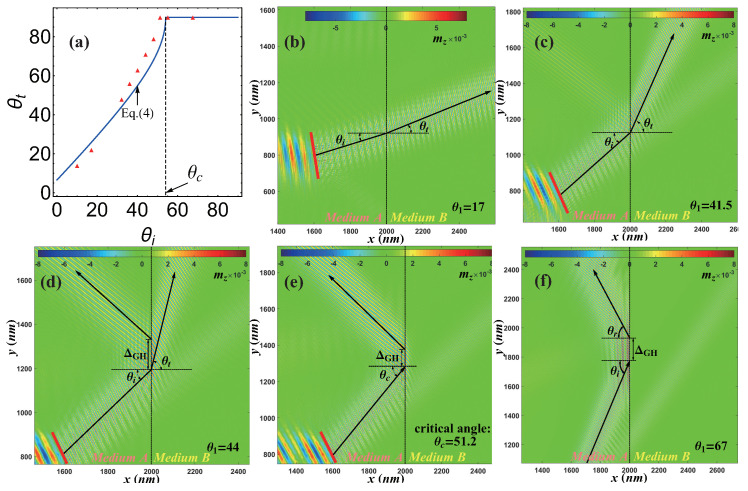
(**a**) The refracted angle as a function of the incident angle. Vertical dashed and solid lines correspond to the critical angle $\theta_{c}$. (**b**–**f**) The micromagnetic simulations results for spin-wave beam reflection and refraction under different incident angles (**b**) $\theta_{i}=17^{\circ }$, (**c**) $\theta_{i}=41.5^{\circ }$, (**d**) $\theta_{i}=44^{\circ }$, (**e**) $\theta_{i}=51.2^{\circ }$ and (**f**) $\theta_{i}=67^{\circ }$. The DMI constants in medium A and B are $D_{1}=4\times 10^{-3}$ J/m${}^{2}$ and $D_{2}=3.5\times 10^{-3}$ J/m${}^{2}$, respectively. The color map shows the z component of the magnetization in the snapshot of micromagnetic simulations at some selected time. The black solid lines correspond to the rays of the incident and refractive beams. The red rectangular area is the excitation area of the spin-wave, and the exciting field frequency is f=100 GHz.

In Figure 3a, we show the refracted angle $\theta_{t}$ as a function of the incident angle $\theta_{i}$ from micromagnetic simulations (red triangle) and the prediction from Equation (4) (blue curve) with DMI constants $D_{1}=4\times 10^{-3}$ J/m${}^{2}$ and $D_{2}=3.5\times 10^{-3}$ J/m${}^{2}$, respectively. The micromagnetic simulation for the five different incident angles, $\theta_{i}=17^{\circ }$, $41.5^{\circ }$, $44^{\circ }$, $51.2^{\circ }$ and $67^{\circ }$, are displayed in Figure 3b–f. Figure 3b–d correspond to the refraction mode, and Figure 3e,f are the total reflection mode. Vertical dashed lines correspond to the interface at x=2000 nm between medium A (left) and medium B (right).

The critical angle observed in our simulation is estimated to be $\theta_{c}=51.2^{\circ }$ as shown in Figure 3e. It is important to comment that the spin-wave propagation direction is not strictly perpendicular to the spin-wave wavefronts in our simulation. That is to say, it is easy to observe strong anisotropy in the propagation of spin waves. Typically, for in-plane magnetized films, spin waves dynamics are anisotropic. This means that iso-frequency dispersion relation lines (IFDRLs, slices of dispersion relations for particular frequencies) are not circular. Therefore, the group velocity and phase velocities (parallel to the wave-vector) are not parallel to each other, since the group velocity direction should be normal to the IFDRLs [78].

Such an intrinsic anisotropy called spin-wave collimation effect is common in ferromagnetic films with the magnetization fixed in the plane of the film by an external magnetic field or a strong in-plane anisotropy [75,76,77]. However, in the present case, the symmetry of the spin-wave dispersion relation is broken due to the presence of DMI, and the wave vector can be shifted from the direction perpendicular to spin-wave wavefronts [70]. This anisotropy decreases with the increasing frequency of the spin-wave but it is still present at the high frequency f=100 GHz assumed in our simulations.

Moreover, a lateral shift ${▵}_{GH}$ of the spin-wave beam is observed at the interface between the reflected and the incident beams, which is called the Goos–H$\ddot{a}$nchen (GH) effect. The GH effect for spin-waves was reported in Refs. [75,76,77,78]. Furthermore, detailed investigations elucidating the role of inhomogeneous DMI on the GH shift in the reflection of the spin-wave at the interface were discussed in Ref. [22].

## 4. Spin-Wave Fiber and Lens

**Figure 4:**
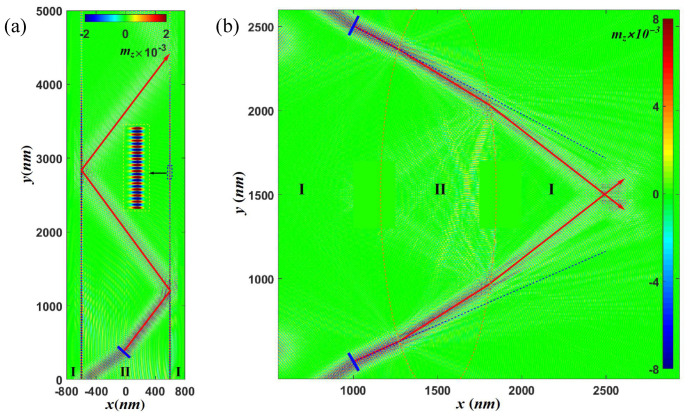
(**a**) Schematic illustration of a spin-wave fiber. The inset shows the enlarged figure at the interface. (**b**) Schematic illustration of a spin-wave convex lens. In all of the above figures, the color map shows z component of the magnetization in the snapshot of micromagnetic simulations at some selected time. The spin-wave trajectories are represented by solid red lines with an arrow. The simulated propagation of the spin wave excited by a AC source in blue bars with an exciting frequency f=100 GHz.

We now turn to the realization of the spin-wave fiber and spin-wave lens, which are important to manipulate spin waves in spin-wave circuitry. Two kinds of spin-wave fiber have been proposed and designed, one based on the TIR by the magnetic domain wall [37] and the other based on the TIR in the medium with a uniform external magnetic field [51]. Here, utilizing the TIR at the interface with a DMI step, we propose a new type of spin-wave fiber as shown in Figure 4a of system size 12 μm × 1.6 μm × 2 nm. The DMI constant in the core (region II, $\left|x\right|\leq 400$ nm) is $0.5\times 10^{-3}$ J/m${}^{2}$ surrounded by transparent cladding FM layers (region I, $\left|x\right|\geq 400$ nm) with a lower index of refraction ($D=-0.4\times 10^{-3}$ J/m${}^{2}$). Two DMI steps are formed with the critical angle $\theta_{c}=48^{\circ }$.

The upper one is located at x=−400 nm and the lower one is located at x=400 nm. In Figure 4a, the spin-wave beam born at the middle of the nanowire (blue bar) propagates inside the core with an incident angle of $52^{\circ }$ greater than $\theta_{c}$. This is different from the unidirectional spin-wave fiber based on domain walls [37]. The fiber here is fully bidirectional for both right/left-moving spin-wave beams when the incident angle is greater than the critical angle.

More interestingly, a bound spin-wave mode propagates a long distance inside the DMI step interface as illustrated in the inset of Figure 4a. Similar to the bound spin wave mode inside a domain wall, which acts as a local potential well for spin waves [31,79], a DMI step also creates an imaginary potential well for the bound spin wave mode [80]. The details will be discussed in our future publications.

A fundamental building block in spin-wave circuitry is a spin-wave lens that can focus or diverge spin-wave beams. Since the dispersion relation strongly depends on DMI constant, we propose a spin-wave lens by tuning the DMI distribution in the film. Figure 4b illustrates an example of a spin-wave convex lens (region I inside red dotted lines) with a DMI constant inside/outside the lens $D=0.5/-0.4\times 10^{-3}$ J/m${}^{2}$, respectively. The size of the sample presented here is 6 μm × 6 μm × 2 nm.

Comparing the solid blue lines along the incident spin-wave beam propagation direction and the spin-wave trajectory (solid red lines with an arrow) passing through the lens, it is easy to observe focusing in the propagation of spin waves. Furthermore, a concave spin-wave lens can be obtained by reversing the DMI constants of regions I and II, which can be used to spit the spin-wave beams. Consequently, we believe that the inhomogeneous DMI can be a good playground to study spin-wave beam propagation [81].

## 5. Conclusions

In conclusion, we both theoretically and numerically studied spin-wave beam propagation in a two-dimensional ferromagnetic film with an inhomogeneous interfacial DMI. Utilizing a spatially varied magnonic refractive index introduced by the variation of DMI, a magnonic metamaterial or graded-index magnonic material can be realized. Snell’s law and TIR for spin waves were predicted with a DMI step interface. Moreover, we designed and studied spin-wave fibers and spin-wave lenses via micromagnetic simulations. We believe that our findings shall open up alternative directions for building reconfigurable, stabilized and scalable spin-wave circuitry in magnon introspection devices.

However, the parameters that we adopted in our simulations to investigate spin-wave propagation in the presence of spatially modulated DMI are not meant to represent a specific material but rather to explore the physical conditions under which the spin-wave total reflection occurs. From the materials standpoint, we acknowledge that the dual requirements of low damping and large DMI may seem incompatible since spin–orbit coupling originating from the adjacent heavy metal layer is detrimental to the former but central to the latter.

The excitation of short-wavelength propagating spin waves with a wavelength of 45 nm in a YIG thin film covered by Co${}_{25}$Fe${}_{75}$ nanowires was reported in a recent experiment [82], where the effective damping was only enhanced to about 10${}^{-3}$. Recent progress in materials science has proven that certain magnetic insulators do possess sizable DMIs either in their bulk [83,84,85] or at the interface [86,87,88]. Although these values remain small (typically $∼10^{-3}–10^{-2}$mJ/m${}^{2}$), these results open interesting perspectives for the achievement of large DMIs in magnetic insulators.

## Data Availability

The data that support the findings of this study are available upon reasonable request from the authors.
